# Continuity and Change in Substance Use Patterns During the Transition from Adolescence to Young Adulthood: Examining Changes in Social Roles

**DOI:** 10.1007/s11469-024-01342-9

**Published:** 2024-06-17

**Authors:** Gabriel J. Merrin, Jennifer A. Bailey, Adrian B. Kelly, Vi T. Le, Jessica A. Heerde, Elizabeth Doery, Ebru A. Batmaz, John W. Toumbourou

**Affiliations:** 1 Human Development and Family Science, Syracuse University, 150 Crouse Dr, Syracuse, NY 13244 USA; 2 Social Development Research Group, School of Social Work, University of Washington, 9725 3Rd Ave NE, Suite 401, Seattle, WA 98115 USA; 3 School of Psychology and Counselling, Queensland University of Technology, 2 George St, Brisbane City, QLD 4000 Australia; 4 Department of Paediatrics, Faculty of Medicine, Dentistry and Health Sciences, The University of Melbourne, Grattan Street, Melbourne, Parkville, Victoria 3010 Australia; 5 Centre for Social and Early Emotional Development, School of Psychology, Deakin University, 221 Burwood Highway, Burwood, Victoria 3125 Australia

**Keywords:** Polysubstance use, Adolescence, Young adult, Developmental transitions, Social role transitions, Multidimensional growth mixture model

## Abstract

This study offers a model for using multidimensional growth mixture models to identify polysubstance use trajectories by examining transitions among conjoint substance use trajectories from adolescence to young adulthood and exploring potential moderators that may facilitate transitions towards healthier substance use trajectories in young adulthood. Longitudinal mixture modeling was used to examine six waves of data collected during adolescence (ages 13, 14, 15) and young adulthood (ages 25, 29, 31) in Seattle, Washington. Data were drawn from the International Youth Development Study, a longitudinal, cross-national study examining the life course patterns of substance use and development among youth. Participants (*N* = 961) completed questionnaires on six occasions that assessed demographics (sex, race, highest parent education), suspension and expulsion, individual substance use, partner substance use, and social role transitions (education, marriage, childbearing, employment). Four substance use classes were identified in adolescence and included *low use* (*n* = 572, 59.6%), *alcohol dominant* (*n* = 177, 18.4%), *increasing use* (*n* = 103, 10.7%), and *poly-use* (*n* = 109, 11.3%). Five substance use classes were identified in young adulthood and included *low use* (*n* = 134, 15.3%), *alcohol only* (*n* = 349, 39.8%), *alcohol and tobacco* (*n* = 97, 11.0%), *alcohol and cannabis* (*n* = 162, 18.5%), and *poly-use* (*n* = 135, 15.4%). The transition from adolescence to young adulthood showed the strongest continuity in the *poly-use* class and the weakest in the *low use* class, with a general trend toward adding substances rather than reducing them. College graduation moderated the transition in substance use patterns from adolescence to young adulthood for *low use* and *alcohol dominant* adolescent classes but not for the *poly-use* class. Delays in adult role assumptions were not consistently associated with substance use classes during this transition. However, where significant, delayed marriage and parenthood acted as protective factors against the progression of substance use leading into young adulthood. The findings underscore the need for early detection and tailored prevention efforts among adolescents. By identifying pivotal periods and specific substance use patterns, these findings inform the timing and focus of targeted interventions designed to reduce the escalation of substance use leading into young adulthood.

Polysubstance use (poly-use) is common in adolescence and has strong continuity leading into young adulthood (Nelson et al., 2015; Tomczky et al., 2016). The onset of poly-use in adolescence is associated with a host of adverse outcomes in both adolescence and adulthood, such as higher levels of academic failure, mental health problems, problematic substance use patterns, involvement with the justice system, and lower financial earnings (Brook et al., 2014; Kelly et al., 2015; Nelson et al., 2015; Vergunst et al., 2022). Poly-users have also been shown to have worse treatment outcomes and higher rates of relapse compared to single-drug users (Staiger et al., 2013). While the need to better understand poly-use across adolescence and young adulthood is evident, heterogeneity in poly-use patterns characterized by differences between people and across time makes modeling changes across developmental transitions challenging. The current study provides a model for using multidimensional growth mixture models (MGMMs) to identify trajectories of poly-use in adolescence and young adulthood. It then investigates transitions between conjoint substance use trajectories from adolescence into young adulthood and examines potential moderators that may promote transitions into healthier substance use trajectories in adulthood.

Previous substance use research has leveraged finite mixture models, such as latent class analysis (LCA; Tomczyk et al., 2016; de Jonge et al., 2022) and growth mixture models (GMMs; Thompson et al., 2018) alongside latent Markov models (i.e., latent transition analysis LTA; Choi et al., 2018; Merrin et al., 2018) to identify use classes and examine changes across adolescence and young adulthood. Although these person-centered methods capture heterogeneity in use patterns and their transitions, the common application of LCA with LTA, where poly-use classes are modeled at each time point and subsequent transitions across time are estimated from wave to wave (lagged effect), fails to capture the longitudinal trajectories (rates of change) across waves. Further, GMM can model longitudinal trajectories, but they restrict the analysis to one substance, thus failing to account for poly-use. These methodological gaps in how poly-use is typically modeled are limited in that they do not adequately capture longitudinal trajectories of poly-use across adolescence and young adulthood. Given the sustained adverse effects of adolescent-onset poly-use leading into adulthood, methods that capture longitudinal trajectories of poly-use across this pivotal developmental transition are needed and have the potential to offer novel insights (Kelly et al., 2015; Nelson et al., 2015; Vergunst et al., 2022). One such method is MGMM, sometimes called parallel-process GMM or group-based multi-trajectory models. MGMM provides a mechanism to estimate longitudinal poly-use trajectories over longer periods of time by simultaneously modeling growth in each substance and then fitting a latent class variable to capture conjoint longitudinal substance use trajectory classes. The application of MGMM in poly-use research enables researchers to simultaneously capture the longitudinal trajectory classes of multiple substances, effectively addressing notable shortcomings in prior poly-use studies.

## Prior Studies Using MGMM

A limited body of research has examined the developmental trajectories of poly-use throughout adolescence and young adulthood utilizing MGMM. Most studies focused on the use of two substances, including alcohol and tobacco (Cance et al., 2017; Jackson et al., 2005), alcohol and cannabis (Hix-Small et al., 2004), and tobacco and cannabis (Cho et al., 2021; Dunbar et al., 2020; Lanza et al., 2021). Two studies included alcohol, tobacco, and cannabis (Brook et al., 2014; Richmond-Rakered et al., 2016), and one recent study examined alcohol, tobacco, cannabis, and illicit drugs (Vergunst et al., 2022). The identified classes typically varied between four and five and included early and late onset as well as increasing and decreasing poly-use trajectories. Of these studies, three—including the only study to include illicit drugs—focused solely on adolescence (Cho et al., 2021; Hix-Small et al., 2004; Vergunst et al., 2022), two focused exclusively on young adults (Brook et al., 2014; Cance et al., 2017), and four captured both adolescence and young adulthood (Dunbar et al., 2020; Jackson et al., 2005; Lanza et al., 2021; Richmond-Rakerd et al., 2016). Notably, two studies (Cho et al., 2021; Richmond-Rakerd et al., 2016) utilized a multiple event process survival mixture model (i.e., parallel survival mixture model) to examine patterns of actual poly-use initiation as the event. Generally, these studies adjusted for several covariates and examined outcomes related to mental health, substance use, and social role transitions. The current study extends this research by examining the trajectories of four substance types (alcohol, tobacco, cannabis, illicit drugs) in both adolescence (ages 13, 14, 15) and young adulthood (ages 25, 29, 31) separately using MGMM. We then further extend prior work by examining transitions between adolescent and young adult trajectories using LTA while controlling for multiple covariates.

## The Influence of Adult Roles on Continuity and Change in Poly-Use Trajectories

The transition from adolescence to adulthood marks a pivotal time characterized by the acquisition of adult roles that have lasting implications for an individual’s financial, social, and familial well-being in adulthood (Waters, 2011). The assumption of adult roles, as marked by social transitions in education (e.g., college graduation), family (e.g., marriage, parenthood), and work (e.g., full-time employment), is associated with decreased substance use in young adulthood (Bachman et al., 2002; Staff et al., 2010). However, shifting social norms have created greater variability in both the timing and sequencing of these social role transitions. Unlike past generations, where normative age-graded milestones that demarcated adulthood were achieved earlier and in sequence, contemporary trends show greater heterogeneity in the pathways to adulthood (Arnett, 2000). Given these shifts and delays in social role transitions leading into adulthood, there is a need for longitudinal research that spans a wider age range to provide a more comprehensive examination during this pivotal developmental transition. Further, the timing and nature of these social role transitions may influence substance use patterns. Thus, the current study examines whether and how transitions in social roles and their timing can moderate the conjoint trajectories of substance use from adolescence into young adulthood.

**Fig. 1:**
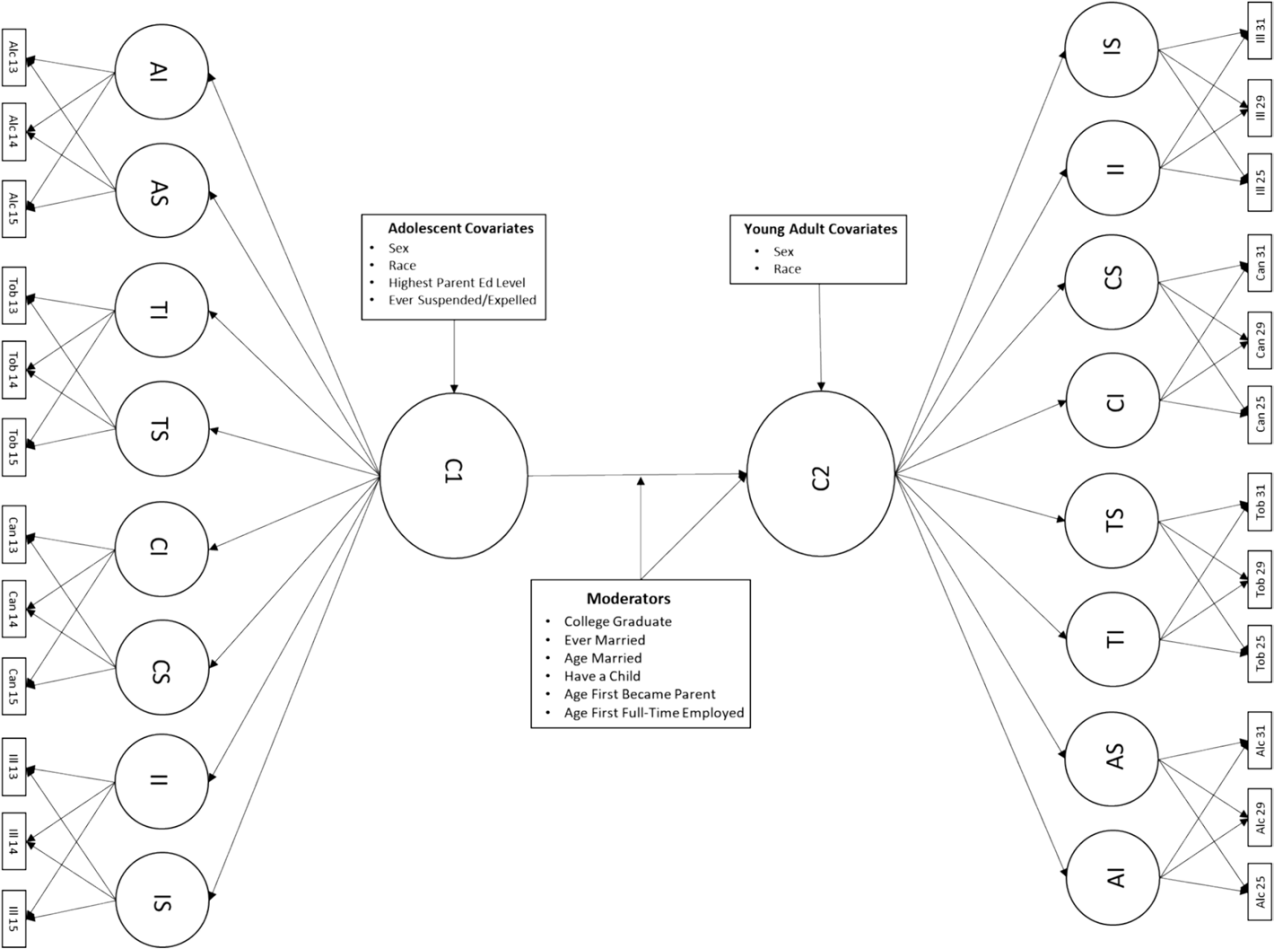
Latent transition analysis between two multidimensional growth mixture models with covariates and young adult moderators. A, alcohol; T, tobacco; C, cannabis; I, illicit drugs. The I and S represent intercept and linear slope parameters. C1 (ages 13, 14, 15) and C2 (ages 25, 29, 31) represent the multidimensional growth mixture class variables. The abbreviations Alc (alcohol), Tob (tobacco), Can (cannabis), and Ill (illicit drugs) followed by the numbers (13–15) denote the use of each substance at ages 13, 14, and 15, respectively. Similarly, Alc, Tob, Can, Ill, followed by the numbers (25–31), denote the use of each substance at ages 25, 29, and 31

In summary, this study adds to the literature by examining (1) the heterogeneity in poly-use trajectories during adolescence and young adulthood; (2) the role of demographic factors, suspension/expulsion experiences, and partner use as predictors of these substance use classes; and (3) the extent to which social role transitions in education, family, and work mitigate risky substance use patterns during the transition from adolescence to young adulthood. See Fig. 1 for the hypothesized multivariate model.

## Methods

### Participants and Procedures

Data were drawn from the International Youth Development Study (IYDS), a longitudinal, cross-national study examining the life course patterns of substance use and development among youth in Washington State (USA) and Victoria (Australia). Participants were initially recruited in 2002 at mean age 13 years (7th grade) and re-surveyed at mean ages 14 and 15 years (adolescence; grades 8 and 9; 2002–2004) and 25, 29, and in the USA only, 31 years (adulthood; 2014–2020). Within each state, a two-stage cluster sampling approach was used to ensure state-wide, representative samples. First, public and private schools were selected using a probability proportional to size sampling procedure. Then, one classroom from each sampled school was randomly selected. Additional information about the IYDS study design, sampling, and methods are available elsewhere (McMorris et al., 2007). The study was approved by the University of Washington Human Subjects Institutional Review Board (USA) and Human Ethics in Research Committees at the Royal Children’s Hospital, Deakin University, and The University of Melbourne in Australia.

Because only two waves of adult data are available in the Victoria subsample, the present analysis was limited to data from participants in Washington. Around 78% of eligible seventh grade students in participating Washington schools were recruited into the study, with nonparticipation partly attributable to nonreturn of consent form (11%) or explicit parental refusal (12%; Bailey et al., 2021). Participant demographics were representative of Washington middle school students in 2002. Adolescent surveys were administered in classrooms, with parental permission obtained for youth under age 18. Young adult surveys were administered online, and respondents provided informed consent at each survey wave.

### Measures

#### Covariates

##### Demographics

Controls included sex (male as the reference), race (white as the reference), and highest parent education (i.e., grade school or less to graduate or professional school).

##### Suspension and Expulsion

Two separate items that assessed the history of school suspension and expulsion during adolescence (13–15) were included and were measured using a dichotomous scale (yes or no).

##### Partner Substance Use

Past 12 months of partner use of alcohol, tobacco, cannabis, or illicit drugs was assessed in young adulthood (ages 25–31) using a dichotomous scale (yes or no).

#### Social Role Moderators

##### College Graduate

College graduation (measured in young adulthood) was assessed using a dichotomous item (yes or no).

##### Married and Age Married

The marital status of participants was evaluated using a dichotomous item. In addition, the age at which participants were first married was measured using a continuous measure of age.

##### Parent and Age First Became a Parent

Parenthood was assessed using a dichotomous item for whether an individual was a parent. The age at which participants first became parents was self-reported using a continuous measure of age.

##### Age of Full-time Employment

Participants self-reported the age at which they first entered full-time employment (continuous). Whether participants ever had a full-time job was not included because most participants (94.7%) reported having full-time employment during young adulthood.

#### Substance Use

##### Adolescence

Self-reported lifetime use of alcohol, tobacco, cannabis, and illicit drugs was assessed at each wave in adolescence. Due to low response rates for some substances and to balance the computational demands of the multivariate models, all substance use items were dichotomized.

##### Young Adulthood

To capture current use patterns, past 30-day use of alcohol, tobacco, cannabis, and illicit drugs in young adulthood was self-reported. To balance the computational demands of the multivariate models, these substance use items were also dichotomized.

### Analysis

Longitudinal data across six waves (*N* = 961) in adolescence (ages 13, 14, 15 years) and young adulthood (ages 25, 29, 31 years) was used. The analysis proceeded in three phases. In phase 1, we fit an MGMM to simultaneously examine conjoint longitudinal trajectory classes of alcohol, tobacco, cannabis, and illicit drugs in adolescence and young adulthood separately (Wickrama et al., 2021). We used MGMM over other mixture models because of its ability to simultaneously model trajectory classes for multiple substances, a feature notably lacking in previous poly-use research. This involved fitting a growth model (intercept and linear slope) for each substance (alcohol, tobacco, cannabis, and illicit drugs) and then estimating a latent class variable to capture the conjoint trajectories of all four substances simultaneously. The models accounted for differences in spacing between waves in young adulthood (i.e., 4-year gap from 25 to 29, 2-year gap from 29 to 31). We completed a class enumeration process for both adolescence and young adulthood, identified the number and composition of the longitudinal trajectory classes, and named them. We used several fit indices: − 2 log likelihood (-2LL), Akaike information criteria (AIC), Bayesian information criteria (BIC), consistent Akaike information criteria (CAIC), Approximate weight of evidence criterion (AWE), and the Lo-Mendell-Rubin adjusted likelihood ratio test (LMRT) with substantive interpretation to select the number of classes. We followed the manual three-step approach, which uses logits to fix individuals in the classes, preventing them from moving when adding predictors and outcomes to the model (Asparouhov & Muthén, 2014). We then examined several predictors of the classes in adolescence and young adulthood to understand the individuals in each class better. In adolescence, we included demographics (sex, race, parent education) and history of being suspended or expelled. In young adulthood, we included demographics (sex and race) and adjusted for partner substance use (alcohol, tobacco, cannabis, illicit drugs). In phase 2, we examined the transition probabilities from substance use classes during adolescence to substance use classes in young adulthood, including controls in the model. In phase 3, we examined the assumption and timing of social roles as moderators of the substance use transitions identified in phase 2 to understand how they may influence transitions into and out of risky use patterns between adolescence and young adulthood. Missing data were rare (91.3% retention); despite this, we used full information maximum likelihood and included demographic variables in our model to adjust for potential bias due to missing data on sex, race, parent education, and history of school suspension/expulsion. All models were run in Mplus 8.7 using maximum likelihood estimation and declared outcomes as categorical.

## Results

### Descriptive

The analytic sample included 961 participants (Table 1). The mean age at baseline was 13.1 years old (standard deviation [S.D.]: 0.44), with 50.9% of participants identifying as female. Most participants reported their race as White (68.9%), with fewer participants reporting their race as Asian (3.9%), Black (3.4%), Native Hawaiian or Pacific Islander (1.6%), Hispanic or Latinx (10.2%), multiracial (7.1%), or Native American (4.9%). By age 15, around 27.4% of participants reported a history of school suspension, while 5.5% reported ever being expelled. Over half (52.4%) of participants had parents who had completed college, graduate, or professional school. By age 31, most participants had not graduated from college (63.7%) and had not been married (55.4%). The majority (57.7%) of participants had their first full-time job before age 19. Partner substance use was commonly reported for alcohol (93.7%), cannabis (56.5%), and tobacco (45.8%), with fewer participants reporting their partner’s use of illicit drugs (23.0%). Fifty percent of participants had a child (mean age at oldest child’s birth: 24.1 years [SD: 4.2]).

**Table 1:** Mean (or *n*) and standard deviation (or %) of all study variables

	Mean (or *n*)	SD (or %)
Baseline age	13.09	0.44
Sex		
Male	472	49.12%
Female	489	50.88%
Race		
Asian	36	3.86%
Black	32	3.43%
Hawaiian	15	1.61%
Latinx	95	10.18%
Multiracial	66	7.07%
Native	46	4.93%
White	643	68.92%
Highest parent education		
Grade school or less	12	1.50%
Some high school	53	6.60%
Completed high school	164	20.42%
Some college	153	19.05%
Completed college	305	37.98%
Graduate or professional school	116	14.45%
Ever suspended from school	230	27.41%
Ever expelled from school	46	5.50%
Individual graduated college		
No	563	63.69%
Yes	321	36.31%
Ever married		
No	532	55.36%
Yes	429	44.64%
Age married	24.30	3.44
Has a biological child		
No	442	50.00%
Yes	442	50.00%
Age first became a parent	24.05	4.15
Age first full-time employed	19.27	2.95
Age 19 or older first full-time employment	336	42.32%
Partner substance use		
Alcohol		
No	51	6.30%
Yes	759	93.70%
Tobacco		
No	439	54.20%
Yes	371	45.80%
Cannabis		
No	352	43.46%
Yes	458	56.54%
Illicit Drugs		
No	624	77.04%
Yes	186	22.96%

### Class Enumeration

**Fig. 2:**
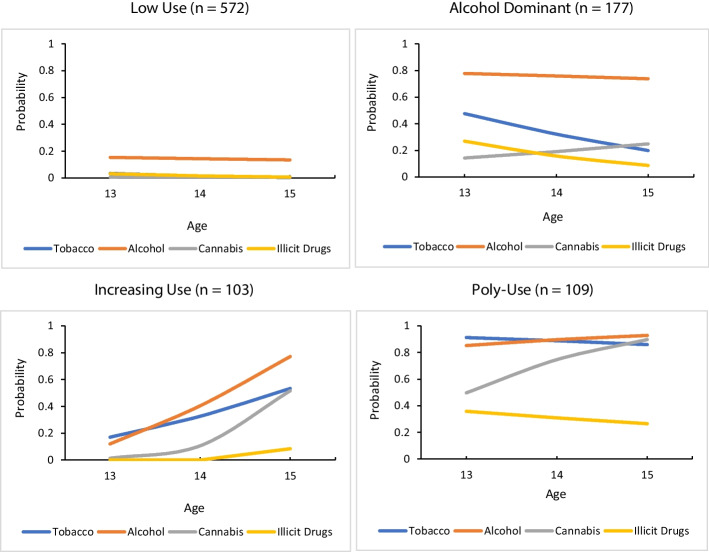
Plotted probabilities of a four class multidimensional growth mixture model of past year tobacco, alcohol, cannabis, and illicit drugs use across ages 13, 14, and 15

We completed a class enumeration for both adolescent and young adult time periods. Starting with a one-class solution, we added classes one at a time and examined the fit indices. The class enumeration process ended when we obtained either a non-significant adjusted LRT value or model non-convergence. Five models (1–5 classes) were fit during adolescence (ages 13, 14, 15), with the four-class solution having the best fit and substantive interpretation (Table 2). The four-class solution had an entropy value of 0.802, suggesting good class separation. The four classes included *low use* (*n* = 572, 59.6%), which was the largest class and had very low endorsement probabilities of all substances; *alcohol dominant* (*n* = 177, 18.4%), which had high, stable rates of alcohol use with lower and decreasing rates of the other substances; *increasing use* (*n* = 103, 10.7%), which showed increasing rates of use of all substances; and *poly-use* (*n* = 109, 11.3%), which showed high rates of use of all substances (except illicit drugs which was low). See Fig. 2 for plotted adolescent substance use classes.

**Table 2:** Fit indices for class enumeration process of multidimensional growth mixture model of past year tobacco, alcohol, cannabis, and illicit drugs use across ages 13, 14, and 15

Classes	− 2LL	AIC	BIC	CAIC	AWE	Adj. LRT	Entropy
Class 1	12,642.67	12,650.67	12,670.14	12,674.14	12,709.61	-	1
Class 2	8624.05	8658.05	8740.80	8757.80	8908.56	0.001	0.878
Class 3	8363.52	8415.52	8542.09	8568.09	8798.65	0.001	0.807
Class 4	8265.00	8335.00	8505.38	8540.38	8850.76	0.01	0.802
Class 5	8191.29	8279.29	8493.48	8537.48	8927.67	0.113	0.769

**Fig. 3:**
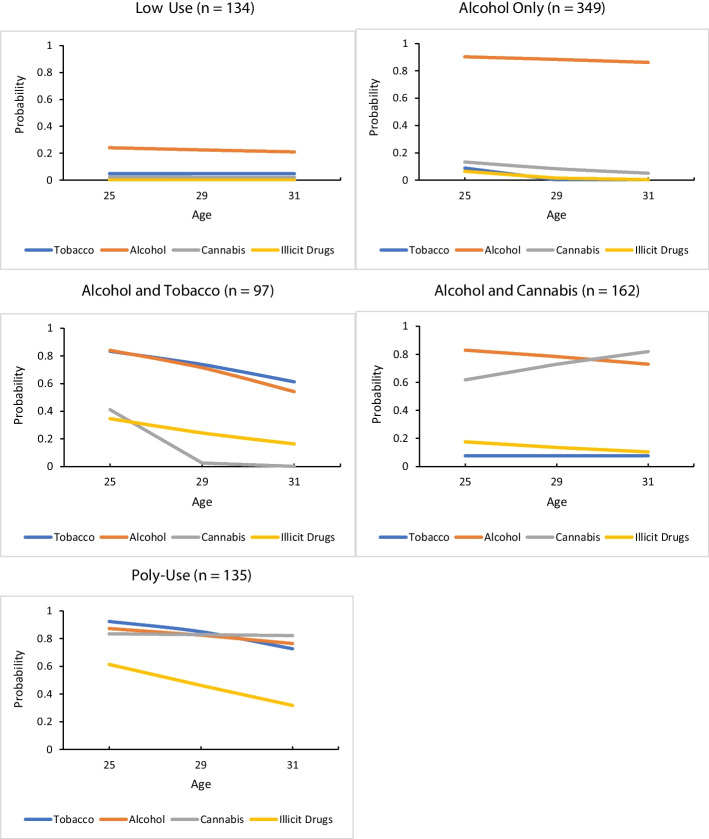
Plotted probabilities of a five class multidimensional growth mixture model of past 30-day tobacco, alcohol, cannabis, and illicit drugs use across ages 25, 29, and 31

A total of seven models (1–7 classes) were fit during young adulthood (ages 25, 29, 31), with the six-class solution having the best fit. Since one of the classes was extremely small (*n* = 35) and was not substantively different from another class, a five-class solution was selected as the final model (see Table 3). The five-class solution had an entropy value of 0.748, suggesting good class separation. The five classes included *low use* (*n* = 134, 15.3%), which had very low endorsement probabilities of all substances; *alcohol only* (*n* = 349, 39.8%), which had high, stable rates of alcohol use with lower rates of use for other substances; *alcohol and tobacco* (*n* = 97, 11.0%), which had high, decreasing rates of alcohol and tobacco use with lower rates of use for cannabis and illicit drugs; *alcohol and cannabis* (*n* = 162, 18.5%), which had high rates of alcohol and cannabis use with lower rates of use for tobacco and illicit drugs; and *poly-use* (*n* = 135, 15.4%), which had high rates of use for all substances (illicit use decreased over time). See Fig. 3 for plotted young adult substance use classes.

**Table 3:** Fit indices for class enumeration process of multidimensional growth mixture model of past 30-day tobacco, alcohol, cannabis, and illicit drugs use across ages 25, 29, and 31

Classes	− 2LL	AIC	BIC	CAIC	AWE	Adj. LRT	Entropy
Class 1	9304.74	9320.74	9358.95	9366.95	9437.17	-	1
Class 2	8236.90	8270.90	8352.10	8369.10	8518.30	0.001	0.778
Class 3	7985.66	8037.66	8161.85	8187.85	8416.03	0.001	0.767
Class 4	7816.50	7886.50	8053.68	8088.68	8395.86	0.001	0.748
Class 5	7711.00	7798.99	8009.16	8053.16	8439.33	0.0011	0.748
Class 6	7642.26	7748.26	8001.42	8054.42	8519.57	0.0138	0.756
Class 7	7597.88	7721.87	8018.02	8080.02	8624.16	1	0.772

Class 6 had a significant adjusted LRT value; however, one class had fewer than 35 people

### Associations with Substance Use Patterns in Adolescence and Young Adulthood

After identifying the number and nature of substance use classes in adolescence and young adulthood, we examined predictors of the classes using multinomial logistic regression. This step allowed us to better understand the characteristics of the individuals in each of the classes. Odds ratios (OR) and 95% confidence intervals (CI) are presented in Table 4 for adolescence and Table 5 for young adult classes. The OR and CI presented refer to the *low use* class as the reference category; however, all comparisons were examined, and all significant differences are reported in the final column. During adolescence, race was not related to class membership; however, compared to males, females had higher odds of being in the *poly-use* class compared to *low use*, *alcohol dominant*, and *increasing use*. Having a parent with higher education was associated with higher odds of being in the *low use* class compared to *alcohol dominant* and *poly-use* and higher odds of being in the *increasing use* class compared to *alcohol dominant*. Individuals who reported a history of school suspension had higher odds of being in the *poly-use* classes compared to *alcohol dominant* and *low use* and higher odds of being in the *alcohol dominant* and *increasing use* class compared to the low use class. Finally, while adjusting for a history of school suspension, individuals who reported a history of school expulsion had higher odds of being in *the poly-use* class compared to *low use*.

**Table 4:** Odds ratios and 95% confidence intervals from multinomial logistic regression model of covariates on substance use growth mixture classes during adolescence

	Poly-use		Increasing use		Alcohol dominant		Low use (reference)	All significant (*p* < .05) class differences
	Odds ratio	95% CI	Odds ratio	95% CI	Odds ratio	95% CI		
Female (sex)	3.80***	[1.96, 7.37]	0.93	[0.51, 1.70]	1.43	[0.88, 2.34]	1	PU > LU, AD, IU
White (race)	0.69	[0.39, 1.24]	0.65	[0.34, 1.22]	0.81	[0.48, 1.36]	1	
Highest parent education	0.76*	[0.60, 0.97]	1.07	[0.78, 1.46]	0.71***	[0.57, 0.89]	1	LU > AD, PU; IU > AD
Ever suspended	9.60***	[5.04, 18.29]	2.78**	[1.41, 5.49]	3.05***	[1.71, 5.45]	1	PU > AD, LU; AD, IU > LU
Ever expelled	5.51**	[1.55, 19.55]	3.65	[0.77, 17.23]	2.77	[0.57, 13.98]	1	PU > LU

Sex is dichotomous with male as the reference category. Race is treated as dichotomous (White and non-White), with non-White as the reference category. Low use is the reference class, although all significant comparisons are included in the *all significant class differences* column

*PU* poly-use, *IU* increasing use, *AD* alcohol dominant, *LU* low use

^*^p < .05; **p < .01; ***p < .001

**Table 5:** Odds ratios and 95% confidence intervals from multinomial logistic regression model of covariates on substance use growth mixture classes during young adulthood

	Poly-use		Alcohol/tobacco		Alcohol/cannabis		Alcohol only		Low use (reference)	All significant class differences
	Odds ratio	95% CI	Odds ratio	95% CI	Odds ratio	95% CI	Odds ratio	95% CI		
Race, sex, education (M1)										
Female (sex)	0.51*	[0.29, 0.91]	0.87	[0.44, 1.73]	0.96	[0.54, 1.69]	0.87	[0.50, 1.50]	1	LU, AO, AC > PU
White (race)	2.52**	[1.34, 4.73]	0.98	[0.49, 1.94]	1.58	[0.86, 2.91]	1.54	[0.86, 2.79]	1	LU, AT < PU
College graduate	0.62	[0.31, 1.24]	0.65	[0.27, 1.57]	1.80	[0.96, 3.37]	3.12***	[1.71, 5.66]	1	LU > AO; AO > PU, AT, AC; AC > PU, AT
Relationship (M2)										
Ever married	0.24***	[0.12, 0.52]	0.45*	[0.21, 0.97]	0.53*	[0.29, 0.98]	1.00	[0.54, 1.85]		LU, AO > PU, AT, AC; AC > PU
Age married	1.19*	[1.03, 1.38]	1.15	[0.96, 1.36]	1.10	[0.98, 1.23]	1.11	[1.00, 1.23]	1	LU < PU
Childrearing (M3)										
Have a child	0.31***	[0.16, 0.62]	0.40*	[0.16, 1.00]	0.26***	[0.13, 0.51]	0.73	[0.40, 1.36]		LU > PU, AT, AC; AO > PU, AC
Age first became a parent	0.91	[0.82, 1.00]	0.83*	[0.72, 0.96]	0.93	[0.84, 1.03]	1.03	[0.95, 1.12]	1	LU > AT; AO > PU, AT
Employment (M4)										
Age first full-time employed	0.84*	[0.73, 0.97]	0.98	[0.83, 1.17]	0.95	[0.86, 1.06]	1.01	[0.91, 1.12]	1	LU, AO > PU
Partner use (M5)										
Partner use of tobacco	9.66***	[3.51, 26.61]	15.11***	[4.85, 47.11]	1.17	[0.48, 2.84]	1.78	[0.80, 3.99]	1	LU, AO, AC < PU, AT
Partner use of alcohol	7.97*	[1.55, 41.05]	2.46	[0.66, 9.16]	5.04*	[1.13, 22.48]	NA	NA	1	LU < PU, AC
Partner use of cannabis	7.91***	[2.97, 21.06]	2.10	[0.81, 5.42]	17.07***	[6.27, 46.49]	2.19*	[1.03, 4.67]	1	LU < PU, AC, AO; AO < PU, AC; AT < PU, AC;
Partner use of illicit drugs	6.30*	[1.52, 26.03]	3.58	[0.82, 15.58]	2.47	[0.62, 9.81]	0.76	[0.18, 3.25]	1	LU, AO, AC < PU; AO < AT, AC

The estimates across models are semi-adjusted and control for sex, race, and college graduates. Sex is dichotomous with male as the reference category. Race is treated as dichotomous (White and non-White), with non-White as the reference category. Low use is the reference class, although all significant comparisons are included in the *all significant class differences* column. The “NA” indicates that the parameter could not be estimated. The predictors were examined by topic area and in five separate semi-adjusted models (M1–M5) that include (1) demographic and education (sex, race, college), (2) relationship (ever married and age), (3) childrearing (have a child and age), (4) employment (age of full-time), and (5) partner use (tobacco, alcohol, cannabis, illicit drugs)

*PU* Poly-use, *AT* alcohol and tobacco, *AC* alcohol and cannabis, *AO* alcohol only, *LU* low use

^*^p < .05; **p < .01; ***p < .001

In young adulthood (Table 5), we examined associations between demographics, social roles, and partner substance use with young adult substance use patterns. Females (compared to males), college graduates (compared to nongraduates), people with children (compared to those without), and people who were married (compared to unmarried) were most likely to be in the *low use* or *alcohol only* classes. Compared to non-white individuals, white young adults had lower odds of being in the *low use* and *alcohol and tobacco* classes compared to the *poly-use* class. In general, obtaining a college degree was associated with lower risk substance use patterns. Specifically, college graduates had higher odds of being in the (1) *low use* class compared to *alcohol only*; (2) *alcohol only* class compared to *poly-use*, *alcohol and tobacco*, and *alcohol and cannabis*; and (3) *alcohol and cannabis* class compared to *poly-use* and *alcohol and tobacco*. Marriage was associated with lower risk substance use patterns, although the timing was generally not related. Specifically, being married was associated with higher odds of being in the (1) *low use* and *alcohol only* classes compared to *poly-use*, *alcohol and tobacco*, and *alcohol and cannabis* and (2) *alcohol and cannabis* class compared to *poly-use*.

Regarding the timing of adult transitions, controlling for whether someone was ever married or not, young adults who reported getting married at older ages had higher odds of being in the *poly-use* compared to the *low use* class. Childrearing and, to some extent, the timing were associated with lower risk substance use patterns. Specifically, having a child was associated with higher odds of being in the (1) *low use* class compared to *poly-use*, *alcohol and tobacco*, and *alcohol and cannabis* and (2) *alcohol only* class compared to *poly-use* and *alcohol and cannabis*. Controlling for whether someone ever had a child, individuals who reported having their first child at older ages had higher odds of being in the *low use* class compared to the *alcohol and tobacco* class and higher odds of being in the *alcohol only* class compared to the *poly-use* and *alcohol and tobacco* classes. Further, individuals who reported having their first full-time job at older ages had higher odds of being in the *low use* and *alcohol only* classes compared to the *poly-use* class.

Partner substance use predicted higher odds of being in riskier substance use classes. Respondents whose partners used tobacco had higher odds of being in the *poly-use* and *alcohol and tobacco* classes compared to the *low use*, *alcohol only*, and *alcohol and cannabis* use classes. Partner use of alcohol was associated with higher odds of being in the *poly-use* and *alcohol and cannabis* classes compared to the *low use* class. Respondents whose partner used cannabis were most likely to be in the *poly-use* or *alcohol and cannabis* use classes and least likely to be in the *low use* and *alcohol only* classes. Partner use of illicit drugs was associated with higher odds of being in the *poly-use* class compared to the *low use*, *alcohol only*, and *alcohol and cannabis* classes and higher odds of being in the *alcohol and tobacco* and *alcohol and cannabis* classes compared to the *alcohol only* class.

### Transitions in Substance Use Patterns from Adolescence to Young Adulthood

Transition probabilities from adolescent to young adult substance use classes are presented in Table 6. Controls included sex, race, highest parent education, history of school suspension or expulsion in adolescence, and sex and race in young adulthood. The *low use* class had relatively low stability, such that only 21% of adolescents transitioned to the *low use* class as young adults. Instead, most of the *low use* class transitioned to the *alcohol only* class (55.2%), followed by *alcohol and cannabis* (18.4%) and *alcohol and tobacco* (5.4%). There was greater stability in the *alcohol dominant* class, such that 37.3% transitioned to the *alcohol only* class in young adulthood. However, most of this class added at least one substance by young adulthood, transitioning to *alcohol and cannabis* (23.3%), *poly-use* (16.8%), and *alcohol and tobacco* (14.1%), with the fewest transitioning to *low use* in young adulthood (8.5%). Fewer than 1% of adolescents in the *increasing use* class transitioned to the *low use* (0.8%) or *alcohol only* (0.0%) classes as young adults. Instead, most transitioned to a use class with multiple substances that included *alcohol and tobacco* (57.2%), *alcohol and cannabis* (21.5%), and *poly-use* (20.5%). The *poly-use* class showed continuity from adolescence to young adulthood, with 58.1% of individuals remaining in the *poly-use* class. Others transitioned to *alcohol and tobacco* (26.5%), *alcohol and cannabis* (10.1%), and *alcohol only* (4.8%), with very few transitioning to *low use* (0.5%).

**Table 6:** Transition probabilities of class trajectories of adolescent substance use to class trajectories of young adult substance use

	Young adult substance use classes (ages 25–31)				
Adolescent substance use classes (ages 13–15)	Poly-use	Alcohol and tobacco	Alcohol and cannabis	Alcohol only	Low use
Low use	0.000	0.054	0.184	0.552	0.210
Alcohol dominant	0.168	0.141	0.233	0.373	0.085
Increasing use	0.205	0.572	0.215	0.000	0.008
Poly-use	0.581	0.265	0.101	0.048	0.005

The adolescent latent class variable controlled for sex, race, highest parent education, and ever suspended or expelled, and the young adult latent class variable controlled for sex and race

### Social Role Moderators of Transitions in Substance Use Patterns from Adolescence to Young Adulthood

As a final step, we tested whether changes in social roles moderated transitions in substance use patterns from adolescence to young adulthood. Given the size and computational demands of the models, we examined moderators in four separate blocks grouped by adult role domain: (1) education (college graduate), (2) marriage (ever and age of marriage), (3) child rearing (ever and age when first became a parent, and (4) employment (age of first full-time employment). All controls from previous models were retained, and adult role moderators were added along with their main effects (see Fig. 1). For the marriage moderator model, we also adjusted for partner use (alcohol, tobacco, cannabis, illicit drugs). The parameters for the increasing class (the smallest class) could not be estimated due to empty cells in the joint distribution of the categorical latent variables. Similarly, some other effects could not be estimated due to empty cells (indicated with *NA*). Low use was the reference category for young adulthood. Estimates for adult role moderators are presented in Table 7.

**Table 7:** Adult role moderators of the transition between adolescent substance use class trajectories to young adult substance use class trajectories

	Poly use		Alcohol/tobacco		Alcohol/cannabis		Alcohol only		Low use (reference)
Adolescent classes	Odds ratio	95% CI	Odds ratio	95% CI	odds ratio	95% CI	Odds ratio	95% CI	
Low use									
College graduate	0.003***	[0.001, 0.03]	0.001***	[0.001, 0.01]	0.001***	[0.001, 0.004]	0.001**	[0.001, 0.05]	1
Ever married	0.43	[0.001, 488.30]	1.93	[0.001, 86,187.2]	0.94	[0.001, 1626.75]	0.11	[0.001, 268.23]	1
Age married	0.26*	[0.07, 0.93]	0.05***	[0.01, 0.28]	0.24*	[0.07, 0.90]	0.31*	[0.11, 0.82]	1
Have a child	NA	NA	NA	NA	NA	NA	NA	NA	1
Age first became a parent	0.82	[0.49, 1.35]	0.11***	[0.07, 0.19]	0.85	[0.60, 1.19]	0.80	[0.24, 2.67]	1
Age first full-time employed	0.72	[0.13, 4.05]	0.35	[0.02, 5.69]	0.44	[0.05, 3.64]	0.40	[0.03, 6.28]	1
Alcohol dominant									
College Graduate	0.01***	[0.001, 0.12]	0.001***	[0.001, 0.002]	0.001***	[0.001, 0.02]	0.001**	[0.001, 0.12]	1
Ever married	0.19	[0.001, 387.24]	NA	NA	0.38	[0.001, 2125.49]	0.10	[0.001, 542.77]	1
Age married	0.28	[0.07, 1.11]	0.02**	[0.001, 0.21]	0.23	[0.05, 1.004]	0.27*	[0.09, 0.80]	1
Have a child	NA	NA	NA	NA	NA	NA	NA	NA	1
Age first became a parent	0.89	[0.56, 1.41]	0.08***	[0.05, 0.13]	0.86	[0.54, 1.36]	0.69	[0.20, 2.43]	1
Age first full-time employed	0.71	[0.12, 4.23]	0.27	[0.02, 3.88]	0.49	[0.06, 4.11]	0.41	[0.03, 6.72]	1
Poly-use									
College graduate	0.16	[0.001, 124.39]	0.28	[0.001, 240.34]	0.009	[0.001, 31.48]	0.02	[0.001, 93.45]	1
Ever married	NA	NA	NA	NA	NA	NA	NA	NA	1
Age married	0.24*	[0.06, 0.94]	0.04***	[0.01, 0.21]	0.14*	[0.03, 0.70]	0.13**	[0.04, 0.47]	1
Have a child	0.001**	[0.001, 0.14]	0.001**	[0.001, 0.09]	0.001*	[0.001, 0.63]	NA	NA	1
Age first became a parent	4.33	[0.87, 22.67]	0.45	[0.72, 2.76]	4.97	[0.92, 26.85]	3.83	[0.49, 29.75]	1
Age first full-time employed	0.69	[0.12, 3.89]	0.43	[0.03, 6.04]	0.43	[0.05, 3.50]	0.40	[0.03, 6.16]	1

Due to empty cells in the joint distribution of the categorical latent variable for the increasing class (the smallest class), the parameters could not be estimated. The adolescent latent class variable controlled for sex, race, highest parent education, and ever suspended or expelled, and the young adult latent class variable controlled for sex and race. Given the computational demands of the model, a full model with all moderators did not converge; we instead examined moderators in four separate models grouped by adult role domain that included education (college graduate), marriage (ever and age), child rearing (ever had a child and age when first became a parent and employment (age of first full-time employment). The marriage model also controlled for partner use (alcohol, tobacco, cannabis, illicit drugs). The “NA” indicates that the parameter could not be estimated

^*^p < .05; **p < .01; ***p < .001

College graduation moderated the transition in substance use patterns from adolescence to young adulthood for *low use* and *alcohol dominant* adolescent classes but not for the *poly-use* class. Specifically, individuals in the *low use* or *alcohol dominant* classes who reported graduating from college had higher odds of transitioning to the *low use* class in young adulthood compared to all other young adult classes (poly-use, alcohol and tobacco, alcohol and cannabis, and alcohol only). Ever being married did not moderate the substance use transitions. However, individuals in the *low use* and *poly-use* classes who reported getting married at older ages had higher odds of transitioning to the *low use* class in young adulthood compared to all other young adult substance use patterns (poly-use, alcohol and tobacco, alcohol and cannabis, and alcohol only). Similarly, individuals in the *alcohol dominant* class who got married later were more likely to transition to the *low use* class compared to the alcohol and tobacco and *alcohol only* classes. Having a child was associated with higher odds of transitioning from the *poly-use* class to the *low use* class compared to all other substances (note, alcohol-only moderator could not be estimated). In addition, the older an individual was when they reported having their first child, the more likely they were to transition from *low use* and *alcohol dominant* classes in adolescence to the *low use* class compared to the *alcohol and tobacco* class in young adulthood. The age of full-time employment did not moderate transitions between patterns of substance use.

## Discussion

The present study utilized MGMM to examine conjoint trajectories of alcohol, tobacco, cannabis, and illicit drugs across adolescence and young adulthood. Our findings showed variability in poly-use patterns and highlighted notable transitions leading into young adulthood. In addition, the study examined how shifts in social roles related to education, family, and work function as potential protective factors, disrupting risky adolescent use patterns during this pivotal developmental transition. These findings align with previous research on the enduring effects of poly-use leading into adulthood and extend them by examining the influence of social role transitions. Moreover, the innovative methodological approach, allowing for the simultaneous modeling of trajectory classes of four substances, offers a valuable model that may be utilized in future studies to further the understanding of poly-use patterns across the life course.

### Patterns and Transitions in Substance Use from Adolescence to Young Adulthood

Findings concur with prior studies showing patterns of substance use are established in adolescence and have strong continuity leading into young adulthood. The poly-use trajectory classes identified in the current study are largely consistent with those found in previous studies using LCA and MGMM (Tomczyk et al., 2016; Vergunst et al., 2022). Notably, the current study builds upon and extends these findings by modeling four substances across the transition from adolescence to young adulthood. The identification of an alcohol and cannabis class in young adulthood is noteworthy and potentially reflects the sample context of participants in Washington State, where recreational cannabis use has been legal since 2012. The variation in substance use trajectory classes in adolescence and young adulthood aligns with existing research suggesting that, for many, adolescence is a period characterized by substance use experimentation (Khurana et al., 2015). In contrast, adulthood appears to be a time of specializing in particular substances (Palmer et al., 2009). Findings from the current study underscore this point by showing how adolescent classes of alcohol dominant, increasing use, and poly-use evolve into more distinct substance use patterns in young adulthood that include alcohol only, alcohol and tobacco, alcohol and cannabis, and poly-use.

Continuity in substance use patterns in the transition from adolescence to young adulthood was the strongest for the poly-use class and weakest for the low use class. Nearly 60% of adolescent poly-users persisted as poly-users into young adulthood, compared to 37% of those in the alcohol dominant class who remained alcohol only users in adulthood and a mere 21% of those in the low use class who remained in the low use class as a young adult. This pattern was partly explained by the fact that the low use class was the largest class in adolescence and the second smallest in young adulthood, reflecting shifts in age-related norms surrounding the legality of alcohol consumption (Brown et al., 2008). These findings align with previous LTA studies and extend them by demonstrating significant instability in the transition of the low use class from adolescence to young adulthood (Merrin et al., 2018).

Transitions in substance use patterns from adolescence to young adulthood showed a tendency to add substances (i.e., forward transition) rather than subtract (i.e., backward transition), a finding in line with previous research (Merrin et al., 2018). For example, 54% of adolescents in the alcohol dominant class transitioned to poly-use, alcohol and tobacco, or alcohol and cannabis. Notably, a substantial majority (99%) of the increasing substance use class transitioned into poly-use, alcohol and tobacco, or alcohol and cannabis classes in adulthood. This transition pattern reflects a consistent trend towards an increasing substance use profile rather than a decreasing one, emphasizing the addition of more substances and the need to identify varying forms of vulnerability and marginalization as well as modifiable protective factors, particularly for high-risk adolescent use patterns.

### Timing of Adult Roles

Delays in adult role assumptions were not consistently related to substance use classes in the transition from adolescence to young adulthood. Where significant transition moderations were found for delayed marriage and parenthood, they acted as protective factors. The older age of first full-time employment, though not a significant moderator of these transitions, was associated with membership in the low use class compared to the poly-use in young adulthood. As such, the current trend toward later assumption of adult roles may not have pronounced effects—either good or bad—on substance use patterns in young adulthood across use patterns (Arnett, 2010). It is also worth highlighting that having a child, regardless of timing, was a consistently protective moderator, particularly for those in the adolescent poly-use class, and was associated with transitioning to a low use class in young adulthood across all substances. This finding supports previous research that finds decreases in substance use following the transition to parenthood (Bachman et al., 2002).

### Protective Role of Education

Graduating college was a consistent protective factor for young adults who were in the low use and alcohol dominant classes but not the poly-use class as adolescents. This was likely due to the small number of adolescent poly-users who graduated from college, which led to low precision and inflated standard errors. Similarly, we also found that the poly-use class had higher odds of reporting a history of school suspension or expulsion, which is a finding that has also been noted in previous research (Evans-Whipp et al., 2015; Hemphill et al., 2012; Kelly et al., 2015). Together, these findings underscore the potential impact that completing high school and transitioning to and successfully finishing higher education may have on fostering healthier substance use patterns as an adult. Consistent with our findings and previous research, riskier use patterns are often found in youth classified as NEET (Not in Employment, Education, or Training; Baggio et al., 2015). Efforts that seek to prevent disengagement and promote well-being (e.g., health-promoting schools, comprehensive school health programs) and that reengage adolescents in education and training for those who may have missed conventional opportunities (e.g., poly-users) could facilitate successful transitions into adult roles.

### Implications for Practice and Policy

Our findings demonstrate that substance use patterns are often established in adolescence and persist into young adulthood. Although existing legislation imposes age restrictions on the use of certain substances, individual substance use patterns may be established well before individuals reach legal age (e.g., age 18, 21). As such, it is critical to implement early and routine substance use screening within healthcare and educational settings to facilitate early detection. Maintaining confidentially during these screenings is essential, as adolescents engaged in substance use behavior are participating in illicit activities. Understanding the motivations and contexts of adolescent-onset is also essential for identifying early risk factors and addressing systemic inequities. For example, our findings indicate that individuals who had been suspended or expelled from school were more likely to engage in poly-use during adolescence. Early identification of these risk factors is a key strategy to help identify and mitigate long-term adverse effects of adolescent-onset substance use.

Prevention and intervention strategies should start early and be tailored to fit the individual’s current use patterns and needs rather than relying solely on abstinence promotion. For example, harm reduction strategies may be more effective for current users, whereas efforts to delay onset may be beneficial for non-users. Involving adolescents in the development of intervention strategies is also important and allows them to take an active role. These strategies should incorporate evidence-based approaches that clearly communicate potential harms alongside protective behavioral strategies that can help mitigate negative consequences. In addition, tailoring efforts based on demographic factors and contextual risks can enhance the relevance and effectiveness of targeted prevention and intervention efforts. Further, given the strong continuity observed in patterns of substance use from adolescence into young adulthood, requiring complete abstinence as a prerequisite for treatment may be counterproductive. A person-centered approach that acknowledges the perceived benefits of use, offers harm reduction strategies, and encourages moderated use could be more beneficial.

Significant life transitions, like graduating from college, play a protective role; however, adolescent poly-users often face higher rates of suspension and expulsion during high school and are less likely to pursue higher education. Efforts to re-engage adolescents who may have missed conventional educational opportunities are needed. Programs designed to re-engage adolescents should offer education and skills training, job placement, and, when necessary, marriage and parenting support that can help equip adolescents and young adults with the skills needed for adulthood. Finally, substance abuse prevention campaigns should address the complexities of poly-use and not just focus on individual substances.

## Future Research

Future research examining poly-use over time should incorporate innovative methods like MGMM to analyze conjoint trajectories of substance use across pivotal developmental transitions. Although studies that examine a single substance while controlling for others enhance our understanding of substance use and abuse, poly-use remains prevalent. Indeed, approximately 45% of participants in this study reported using more than one substance in young adulthood. Research that isolates single substances may incorrectly attribute harm to those substances alone, thereby overlooking the combined effects of multiple substances. To date, few studies have directly modeled conjoint trajectories of multiple substances over time while also considering predictors and long-term outcomes. To our knowledge, this study is among the first to directly assess protective moderators in the transition from adolescence to young adulthood. MGMM offers great potential for researchers to longitudinally examine poly-use while incorporating predictors, outcomes, and moderators. Future research should adopt similar methods that enable the modeling of conjoint substance use trajectories rather than controlling for specific substances. In addition, identifying early risk factors and modifiable protective factors that can disrupt high-risk substance use patterns leading into young adulthood remains a crucial area of research. Given the strong continuity in use patterns from adolescence to young adulthood found in most poly-use studies, factors such as college graduation that help facilitate a successful transition to adulthood may help individuals move towards safer substance use behaviors in adulthood.

### Limitations

The current study has several limitations. While capturing almost 20 years, the study design was limited to three measurement occasions during adolescence and three during young adulthood, limiting the number of assessments. Due to the computational complexity of the models, the substance use items were dichotomized, which may lead to information loss. Although MGMM offers a novel approach to modeling poly-use longitudinally, its constraints on the class numbers across substances could potentially hide variability since certain substances may have more or fewer classes. The *Increasing* class was relatively small, thus preventing the estimation of adult role moderators specific to this class. The study design also restricted the assessment of directionality (i.e., causality) in patterns of substance use and adult roles. Further, the examination of several predictors and moderators led to a large number of significant tests. The reliance on self-report data among a non-diverse sample from a state where cannabis is legal limits the generalizability of the findings. Washington State was the first state in the USA to legalize cannabis, opening the first legal cannabis dispensary in 2014. This may have influenced the findings, as legalization and subsequent availability of cannabis could have affected the rates of cannabis use observed in the sample. Despite these limitations, the study has several strengths. The prospective longitudinal design, spanning ages 13 to 31 within a cohort recruited as state-representative, and the use of innovative analytic methods provided valuable insights into prevalence, continuity, and change in patterns of substance use across a pivotal developmental transition. To address these limitations, future research should use datasets that include more than three waves of data during both adolescence and young adulthood. Larger samples would allow for a greater number of individuals to potentially transition between classes, thus increasing the precision of the estimates for the social role moderators. Finally, incorporating more diverse samples is crucial for a more comprehensive understanding of the development and progression of poly-use during the pivotal transition from adolescence to young adulthood.

## Conclusion

The current study provided a comprehensive examination of patterns of poly-use from adolescence to young adulthood and the protective nature of social roles utilizing an innovative MGMM that uncovered distinct trajectories and transitions. This analysis spans ages 13 to 31, providing a detailed understanding of the developmental course of substance use while highlighting the continuity and variability in poly-use patterns in the transition from adolescence to young adulthood. By identifying key transitions in education, family, and work roles that act as potential protective factors, the study adds to the knowledge base on how social roles can disrupt risky adolescent use patterns leading into young adulthood, offering potential strategies for targeted intervention. Further, the innovative methodological approach provides a model for future research on poly-use, extending previous research and contributing valuable insights for future research in this area. Together, the findings underscore the importance of early detection and prevention among adolescents by informing the timing and focus of targeted interventions aimed at reducing substance use escalation into young adulthood.

## Disclaimer

The funding agencies had no role in the design of the study, collection, analysis, or interpretation of data, the writing of this report, or the decision to submit this manuscript for publication. Conclusions reflect the points of view of the authors and not the funding agencies.

## Data Availability

Due to privacy restrictions, the study’s data are not publicly available, but scripts and output of the study findings are available upon request.
